# Development and treatment of two distinct pseudoaneurysms following transradial coronary catheterization in a patient with high bleeding risk: a case report

**DOI:** 10.1186/s13256-019-2205-6

**Published:** 2019-09-08

**Authors:** Lingling Wu, Faris Haddadin, Joshua Berookhim, Justin Ratcliffe, Joseph Puma

**Affiliations:** 1 Department of Medicine, Mount Sinai St. Luke’s and Mount Sinai West Hospital, 1111 Amsterdam Ave, New York, NY 10025 USA; 2 Mount Sinai Heart, Mount Sinai St. Luke’s and Mount Sinai West Hospital, 1111 Amsterdam Ave, New York, 10025 NY USA

**Keywords:** Pseudoaneurysm, Coronary artery disease, Intervention, Complication, Compression

## Abstract

**Background:**

A pseudoaneurysm is a rare but serious complication after transradial coronary catheterization. Although different treatment modalities have been proposed to treat post-catheterization pseudoaneurysm, only limited data are available to guide the treatment approach.

**Case presentation:**

We report a rare case of two distinct pseudoaneurysms complicating transradial percutaneous coronary intervention in a 75-year-old Hispanic woman being treated with dual antiplatelet agents for myocardial infarction and warfarin for atrial fibrillation. The pseudoaneurysms were diagnosed with ultrasound and were successfully treated with a series of Terumo Corporation (TR) band compressions.

**Conclusions:**

This case demonstrates the efficacy of compression therapy in managing post-catheterization radial artery pseudoaneurysm in patients with high bleeding risk.

## Background

The radial artery has largely replaced the femoral artery as the first choice for cardiac catheterization access because of its superior safety profile [1]. While pseudoaneurysms are a serious complication of transfemoral access, they occur with much less frequency through the transradial approach. Their clinical implications and treatment options are not well studied [1]. Typically, in the event of a pseudoaneurysm occurrence in any arterial vessel, treatment options include compression therapy, surgical repair, and, more recently, ultrasound-guided thrombin injection [2]. In this case report, we describe the occurrence and treatment of two distinct radial artery pseudoaneurysms following cardiac catheterization in a patient with high bleeding risk. To the best of our knowledge, this is the first case report of two simultaneously developed pseudoaneurysms complicating transradial artery catheterization.

## Case presentation

A 75-year-old Hispanic woman with extensive comorbidities including coronary artery disease, end-stage renal disease on dialysis, paroxysmal atrial fibrillation on warfarin therapy, presented with exertional chest pain for 1 month. Her chest pain was episodic, lasted for a few minutes, was triggered by activities, and alleviated by resting; it was associated with shortness of breath and leg swelling. She takes amlodipine 5 mg daily, losartan 25 mg daily for her hypertension, carvedilol 25 mg twice daily, aspirin 81 mg and atorvastatin 80 mg daily for coronary artery disease, as well as warfarin 6 mg daily for atrial fibrillation. She has a surgical history of abdominal aortic aneurysm repair, cholecystectomy, and arteriovenous fistula on her left arm. Her family history is positive for hypertension and diabetes. She was a former smoker of tobacco of half a pack a day for 30 years. She drinks alcohol occasionally. She was a housewife and lives with her son.

**Fig. 1:**
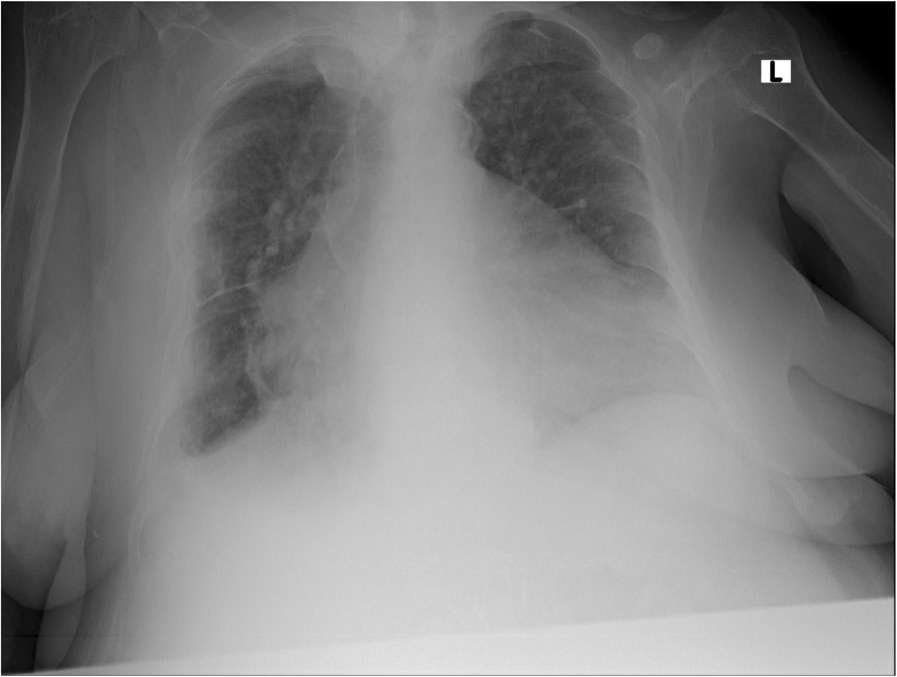
Chest X-rays on admission: mild pulmonary vascular congestion with no focal consolidation

On admission, she had normal temperature of 36.89 ºC (98.4 ºF), elevated blood pressure of 155/75 mmHg, pulse rate of 73 per minute, respiratory rate of 19 per minute, and saturation of 95% on room air. A physical examination was significant for bibasilar lung crackles and bilateral lower extremity pitting edema. A neurologic examination was unremarkable. A complete blood count showed hemoglobin of 6.9 g/dL with normal white cell count and platelets. A basic metabolic panel revealed elevated creatinine of 3.78 and was otherwise unremarkable. Troponin was noted to be mildly elevated at 0.051 ng/mL, which normalized the next day. Brain natriuretic peptide (BNP) was also elevated at 1908.7 pg/mL. An electrocardiogram demonstrated normal sinus rhythm with a rate of 70 beats per minute. A chest X-ray showed mild pulmonary vascular congestion with no focal consolidation (Fig. 1). A transthoracic echocardiogram was performed which revealed an ejection fraction of 55% with no evidence of focal wall motion abnormality.

A presumptive diagnosis of unstable angina was made and a transradial cardiac catheterization was planned. Warfarin was held before the catheterization and pre-procedure international normalized ratio (INR) was found to be 1.7. Establishment of access to her right radial artery was attempted with the use of ultrasound guidance and was successfully performed on the first attempt. However, she was noted to have a slight radial artery spasm during catheter manipulation. She was found to have 70–80% stenosis in distal left coronary circumflex artery and percutaneous transluminal coronary angioplasty using Guidezilla™ (Boston Scientific) 5 French catheter was performed. A Synergy™ (Boston Scientific) drug-eluting stent was then successfully placed. The procedure was not complicated by any difficult access or accidental catheter advances. A TR band (Terumo Corporation) was applied at the puncture site for 5 hours after the procedure and was then removed. Warfarin was resumed after the procedure and dual antiplatelet therapy of aspirin 81 mg daily and clopidogrel 75 mg daily was also started.

**Fig. 2:**
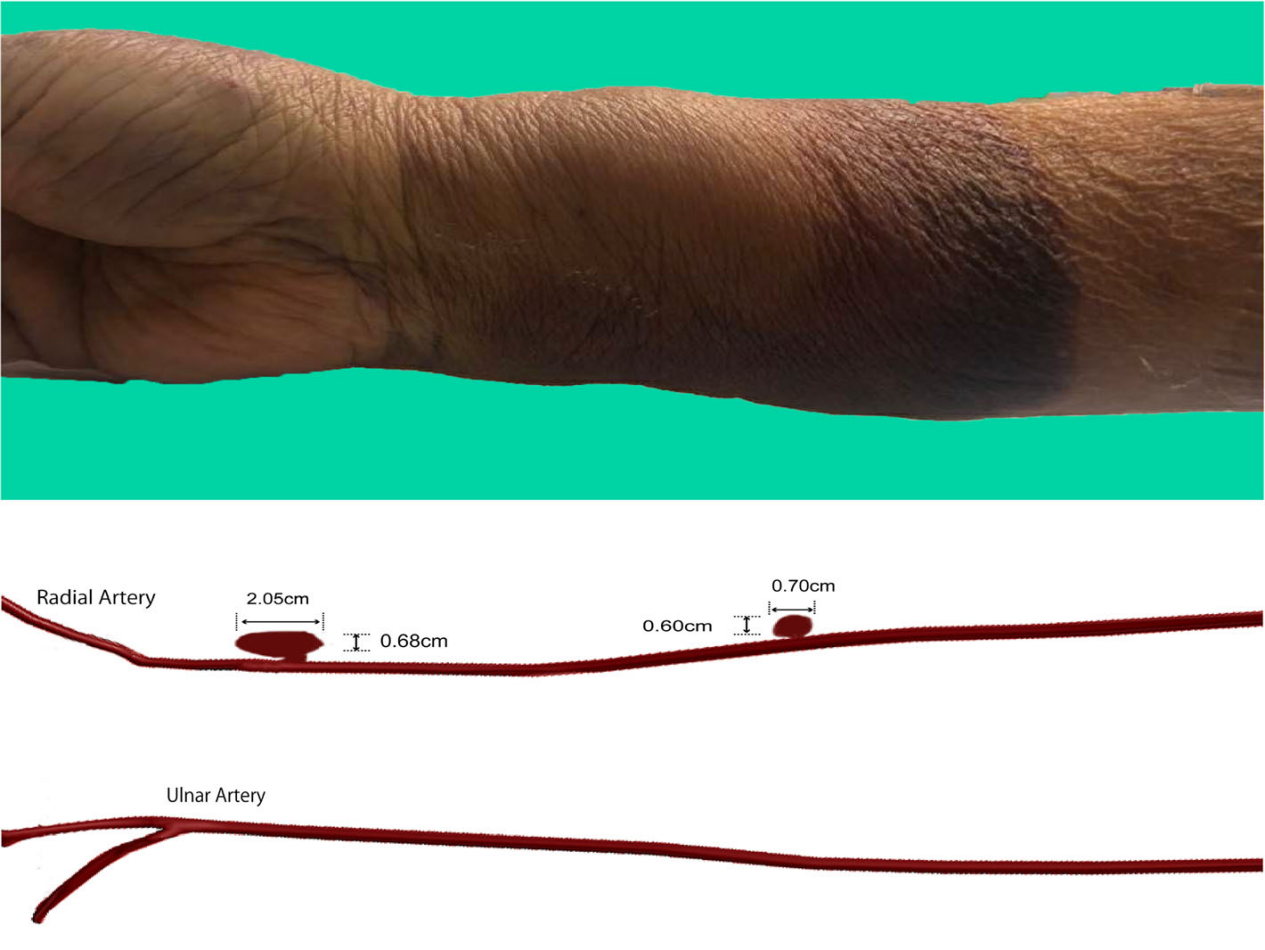
*Upper panel*: Day 2 after percutaneous coronary intervention, the right forearm showed swelling proximal to the puncture site. *Lower panel*: illustration of the location of pseudoaneurysms; the large distal pseudoaneurysm measured at 2.05 × 0.68 × 0.25 cm located near the puncture site, whereas the smaller right mid-forearm pseudoaneurysm measured at 0.70 × 0.60 × 0.09 cm located at the mid-forearm emanating from the radial artery

**Fig. 3:**
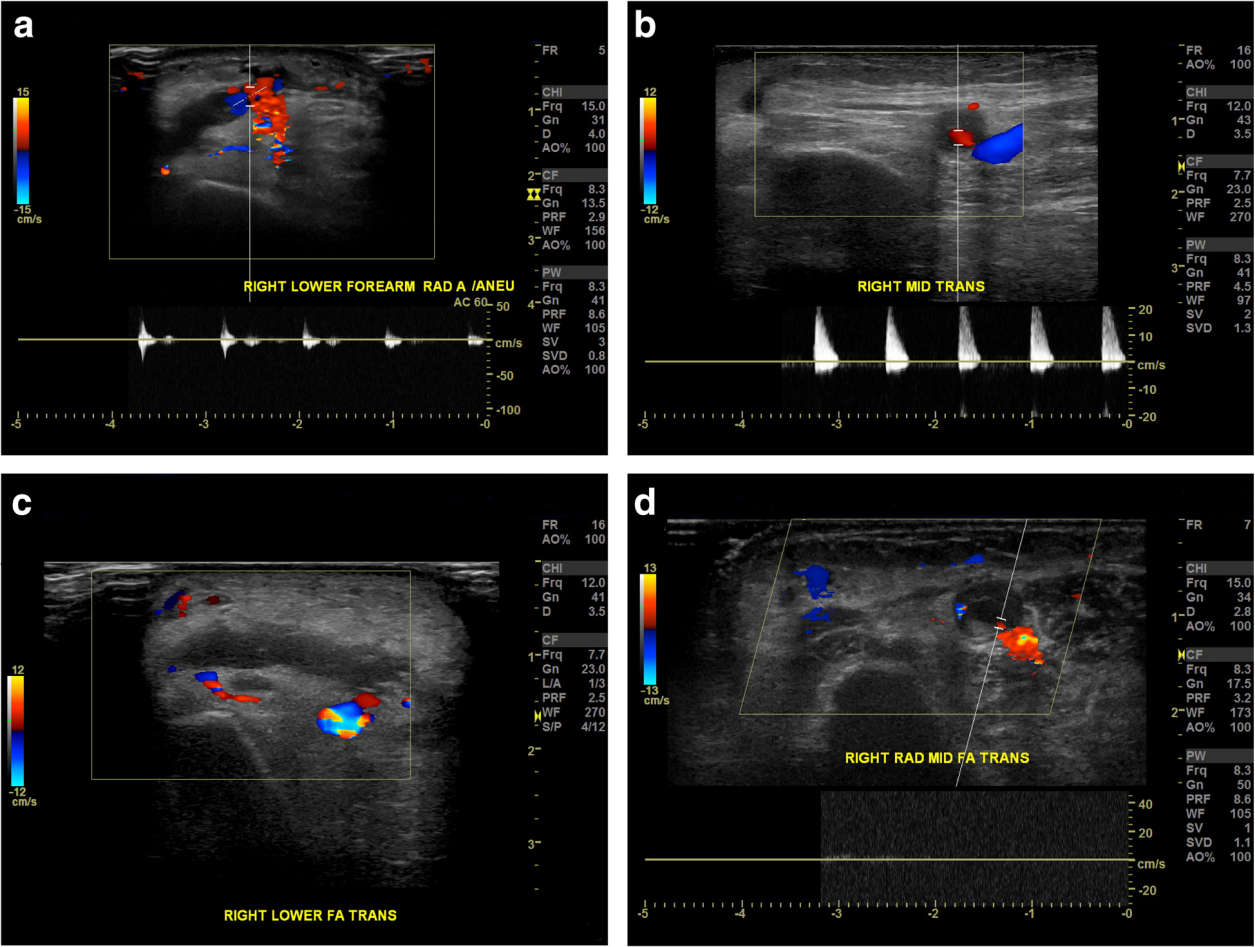
**a** Day 2 after percutaneous coronary intervention, duplex ultrasonography demonstrated right lower forearm pseudoaneurysm with typical “to-and-fro” waveform detected at sac neck that is consistent with a pseudoaneurysm. **b** Day 2 after percutaneous coronary intervention, duplex ultrasonography demonstrated right mid-forearm pseudoaneurysm with positive and negative blood flow shown on duplex Doppler. **c** After 2 days of TR band compression, duplex ultrasonography of the right lower forearm showed complete thrombosed pseudoaneurysm with no color Doppler signals inside. **d** Four days after discharge, duplex ultrasonography of the right mid-forearm showed completely thrombosed pseudoaneurysm

No sign of bleeding or swelling was noticed during the first day after the procedure. On the second day after the procedure, our patient developed significant right forearm swelling (Fig. 2). An examination revealed a hard swollen mass that was tender to palpation. No bruits were appreciated. The swelling was initially treated with bandage compression, but there was no improvement. Doppler ultrasound was then performed which demonstrated a pseudoaneurysm from the radial artery located at the puncture site. The pseudoaneurysm measured 2.05 × 0.68 cm, with a neck size of 0.25 cm (Fig. 3a). A second more proximal pseudoaneurysm at the mid-forearm was also noted with a size of 0.70 × 0.60 cm and a neck size of 0.09 cm (Fig. 3b).

**Fig. 4:**
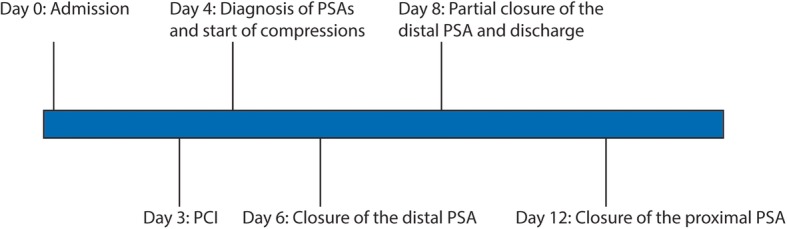
Timeline of the events. PCI percutaneous coronary intervention, PSA pseudoaneurysm

Vascular surgery was consulted for possible surgical intervention. Our patient was deemed to be a poor surgical candidate for surgical repair due to her comorbidities. A trial of compression therapy was recommended. Starting the second day after percutaneous coronary intervention (PCI), TR band compression was reapplied daily with the guidance of ultrasound. Each session lasted 2 hours and TR band was then removed. Distal pulse oximetry and Doppler ultrasound were obtained to ensure the patency of the radial artery during compression. Warfarin was held after the discovery of the pseudoaneurysms. Dual antiplatelet therapy was continued given the presence of coronary drug-eluting stent. A series of vascular Doppler ultrasounds were performed to evaluate if the pseudoaneurysm was still present. The treatment was well tolerated by our patient. After 2 days of TR band compression, the distal pseudoaneurysm thrombosed completely (Fig. 3c). After 4 days of daily TR band compression, the proximal pseudoaneurysm was noted to be partially thrombosed. She was discharged with a plan of out-patient follow-up with vascular surgery. Four days after discharge, the proximal pseudoaneurysm was found to have spontaneously thrombosed during the out-patient visit (Fig. 3d). No surgical intervention was required or performed. Warfarin was eventually resumed after closure of both pseudoaneurysms. A follow-up visit 7 months after discharge showed no recurrence of pseudoaneurysm. A timeline of the events is shown in Figure 4.

## Discussion and conclusions

In this report, we, for the first time in the literature, describe a rare case of two simultaneously developed pseudoaneurysms following a transradial catheterization. We also report using an extrinsic compression device to treat radial pseudoaneurysm in patient with high bleeding risk.

Pseudoaneurysm of the radial artery following transradial coronary angiography and PCIs is generally rare and occurs in approximately 0.009 to 0.1% of cases of cardiac catheterization [2, 3]. Risk factors for pseudoaneurysm formation after coronary angiography include older age, longer catheterization duration, anticoagulants or antiplatelet use, larger sheath diameter, bleeding disorder, and incomplete hemostasis [2]. Despite using a small size catheter, our patient has multiple risk factors for developing pseudoaneurysms, which helps explain why this patient developed multiple pseudoaneurysms after PCI. Post-catheterization pseudoaneurysms are usually associated with the vascular access site, indicating iatrogenic injury played an important role. By contrast, the occurrence of a pseudoaneurysm complication more proximal to the access site is exceedingly rare [4]. In this case, our patient was noted to have a slight radial artery spasm which probably resulted in vessel injury from catheter advancement. Although the ability to advance the catheter past the spasm to complete the procedure usually does not have any clinical manifestations, this patient’s high bleeding risk probably contributed to the development of the proximal pseudoaneurysm after a typically minor iatrogenic injury to the vessel. Although it is not clear whether a radial pseudoaneurysm poses the same limb-threatening risk as a femoral pseudoaneurysm, failure to detect a pseudoaneurysm can sometimes result in spontaneous rupture or infection [5].

The modalities of treatment of arterial pseudoaneurysms include noninvasive methods such as mechanical compression and other invasive approaches such as ultrasound-guided thrombin injection and surgical repair [3]. These treatment modalities largely came from case reports or case series, and the selection of treatment typically depended on the size and the location of pseudoaneurysms. For superficial pseudoaneurysms and small pseudoaneurysms, extrinsic compression can be used [6], whereas for deeper, larger, and more complex pseudoaneurysms, ultrasound-guided thrombin injection or surgical repair should be considered after consulting vascular surgery [7]. In this case, our treatment options were limited by our patient’s bleeding risk and comorbidities. After weighing the benefits against the risks, we eventually chose to start with a trial of extrinsic compression therapy before pursuing any other option. It is worth mentioning that surgical repair remains the most effective treatment of pseudoaneurysm. Should the aneurysm remain patent after TR band compression, a surgical consultation should be obtained. Even in patients that are at higher surgical risk, a surgical repair under local anesthesia can sometimes be achieved [7].

Using compression therapy to treat pseudoaneurysm has existed since the beginning of PCI. Most of the experience on the treatment of arterial pseudoaneurysms using compression therapy came from femoral artery pseudoaneurysms. Ultrasound-guided compression repair (UGCR) has been proposed as an effective treatment for femoral artery pseudoaneurysm with a success rate of 95% [8]. In contrast, compression therapy for radial artery pseudoaneurysm is less well structured. Different instruments have been used for compression including bandage, UGCR, and, more recently, the TR band [6, 9]. Because of its wide availability and simplicity, the TR band has been proposed as an ideal tool for the treatment of radial pseudoaneurysm. Protocols used in these reports vary from case to case, with compression time ranging from 20 minutes to 4.5 hours to even 72 hours [6, 9]. In this case, we chose daily intermittent compression sessions as our approach, assisted by Doppler ultrasound to assess its effectiveness. We found this approach was well tolerated by our patient. We also temporarily held warfarin therapy because of the anecdotal report of a lower success rate of compression therapy for radial artery [10] pseudoaneurysms in patients who receive anticoagulation therapy.

Interestingly, our case showed the size of the pseudoaneurysm does not correspond to the response to treatment. Although the proximal pseudoaneurysm was smaller, it required six sequential treatments session with TR band before it completely thrombosed. A possible explanation is that the proximal pseudoaneurysm is deeper in its anatomic location. Therefore, it may require more time/pressure for extrinsic compression to be effective, suggesting that the effectiveness of this approach may also depend on the location of the pseudoaneurysm.

In conclusion, we report a rare case of two iatrogenic pseudoaneurysms that developed simultaneously after a transradial cardiac catheterization in a patient taking a dual antiplatelet agent and anticoagulation. Extrinsic compression was shown to be well tolerated and effective in this case. TR band compression may be considered a treatment option for radial pseudoaneurysm in patients with high surgical risk.

## Data Availability

All relevant data is contained within the manuscript.

## Abbreviations

BNP: Brain natriuretic peptide
INR: International normalized ratio
PCI: Percutaneous coronary intervention
UGCR: Ultrasound-guided compression repair
